# Cross-cultural adaptation and psychometric validation of the Female Sexual Function Index-6 (FSFI-6) Bangla version

**DOI:** 10.1093/sexmed/qfae044

**Published:** 2024-07-11

**Authors:** Refat Uz Johra, Mohammad Shamsul Ahsan, Ahsan Aziz Sarkar

**Affiliations:** Department of Psychiatry, Sher E Bangla Medical College Hospital, Band road, Barishal 8200, Bangladesh; Department of Psychiatry, Bangabandhu Sheikh Mujib Medical University, Shahbag, Dhaka 1000, Bangladesh; Pabna Mental Hospital, Hemayetpur, Pabna 6602, Bangladesh

**Keywords:** Female Sexual Function Index–6, Female Sexual Function Index–6 Bangla version, female sexual dysfunction

## Abstract

**Background:**

The 6-item Female Sexual Function Index (FSFI-6) is the shortened version of the widely used 19-item FSFI-19, designed for efficient screening of female sexual dysfunction in outpatient settings. However, this shorter FSFI-6 tool has not yet been validated for use in Bangladesh.

**Aim:**

The purpose of this study was to culturally adapt and validate the FSFI-6 in Bangla.

**Methods:**

The FSFI-6 was translated into Bangla using standard adaptation protocols. We interviewed 100 married, sexually active women aged 18 years and over from the outpatient and psychiatric sex clinic of a psychiatry department. Of these women, 50 were clinically diagnosed with sexual disorders based on the *Diagnostic and Statistical Manual of Mental Disorders*, 5th edition, criteria. After obtaining written informed consent, participants completed a semi-structured questionnaire to provide sociodemographic information and the Bangla-adapted version of the FSFI-6. We assessed reliability and construct validity using the Statistical Package for Social Sciences, version 25, along with Classical and Bayesian Instrument Development software.

**Outcome:**

Study outcomes were internal consistency, factor structure, and sensitivity and specificity.

**Results:**

The study involved 100 participants with a mean ± SD age of 30 ± 5.4 years, ranging from 18 to 48 years. The majority of respondents (54.34%) reported issues related to sexual desire. The overall mean score on the Bangla-adapted FSFI-6 was 18.4 ± 5.4. Reliability analysis showed a high internal consistency, with a Cronbach’s alpha of 0.887 indicating robust reliability. Both inter-item correlations and item-total correlations were within the acceptable range. A cutoff value of 19 for the FSFI-6 demonstrated high discriminative power, effectively distinguishing between individuals with sexual disorders and those without sexual disorders or with other psychiatric conditions. The sensitivity at this cutoff was 96%, with a specificity of 100%.

**Clinical Implications:**

The FSFI-6 Bangla version can be used to screen patients for female sexual dysfunction in an outpatient setting.

**Strengths and Limitations:**

The internal consistency of this study, indicated by a Cronbach’s alpha of 0.887, was robust. The instrument is time efficient, user friendly, and well suited for outpatient settings. However, the sampling technique utilized was nonrandomized, confined to a single institution, and did not incorporate assessments for concurrent validity or test–retest reliability.

**Conclusion:**

The FSFI-6 Bangla version showed good reliability and validity in this study, supporting its usability as a valuable tool for screening sexual dysfunction in female.

## Introduction

Female sexual dysfunction (FSD) is a much-discussed topic in various medical fields, with prevalence rates ranging from 30% to 50% across different age groups.^1^^,^^2^ The condition encompasses three primary domains: female sexual interest/arousal disorder, orgasmic disorder, and genito-pelvic pain/penetration disorder.^3^ Quality of life can be substantially affected by FSD, not only for the women who experience it but also for their partners, highlighting its broad impact.^4^ The adverse effects of FSD are felt in both physical and emotional spheres, contributing to social disruption for many women.^5^ Studies indicate a bidirectional relationship between FSD and quality of life throughout a woman's reproductive lifespan and beyond.^4^ A meta-analysis has revealed a strong correlation between sexual dysfunction in women and sexual dysfunction in their male partners, showing a 3-fold increase in male sexual dysfunction among men in relationships with women affected by FSD.^6^ Male partners of women with FSD have a 4-fold higher likelihood of experiencing erectile dysfunction and a doubled likelihood of premature ejaculation.^6^

Despite the significant impact of FSD on quality of life and relationships, research and data on the subject remain limited, and FSD is often underdiagnosed. The diagnostic challenge is compounded by societal and cultural factors that discourage open discussions about sexual health, particularly in conservative Muslim countries like Bangladesh.^7^ In many Asian cultures, including Bangladesh, conversations about sexual health are taboo, leading to reluctance among women to discuss sexual concerns with healthcare providers.^8^ This cultural reticence contributes to underreporting and a lack of diagnosis for FSD. Several psychometric tools have been developed to assess FSD, such as the Female Sexual Function Index–6 (FSFI-6), the Arizona Sexual Experiences Scale, the Derogatis Interview for Sexual Functioning, the Sexual Satisfaction Scale for Women, the Sexual Function Questionnaire, and the Golombok Rust Inventory of Sexual Satisfaction. However, none of these assessment tools are available in Bangla, further hindering accurate diagnosis and research in Bangladesh.

To address the diagnostic challenges posed by FSD, the FSFI has proven to be a valuable tool. As a multidimensional self-report psychometric instrument, the FSFI is widely regarded as the gold standard for evaluating female sexual function.^9^ The FSFI is extensively used for both research and clinical applications, particularly in outpatient settings. However, the structure of the original comprehensive FSFI may be overly detailed for routine clinical use, which prompted the development of an abbreviated version, the FSFI-6. The FSFI-6 is a streamlined version of the original index designed to be shorter, simpler, and less time-consuming, thus making it more practical for patient self-administration. The FSFI-6 comprises six items, each representing one of the six domains of FSD, allowing for a broad yet concise assessment. Despite the reduced length, the FSFI-6 retains the psychometric robustness and reliability of the original FSFI.^10^ This shortened version has been validated in multiple cultures and languages, including Spanish,^11^ Korean,^12^ Portuguese,^13^ Iranian,^14^ and Turkish^15^ cohorts, with a diagnostic cutoff point of 19 established for identifying FSD.

In Bangladesh, the development of sexual health services is limited, and the societal restraint often leads to hesitation and discomfort in addressing FSD. Given this context, a self-reported screening tool like the FSFI-6 could serve as an effective method for assessing FSD among Bangladeshi women in outpatient settings. However, prior to this study, the FSFI-6 had not been culturally adapted or validated in Bangladesh. The primary objective of the research was to evaluate the reliability and validity of the Bangla version of the FSFI-6, thereby providing a culturally appropriate tool for rapid screening of FSD. By validating this abridged questionnaire, the researchers aimed to lay the groundwork for more extensive research and improve the identification and treatment of FSD in Bangladeshi women.

## Methods

### Participants

The study included 100 Bangla-speaking married women aged over 18 years who had stable sexual involvement with a male partner for at least 4 weeks. The participants were seeking psychiatric consultation at the Outpatient Department and Psychiatric Sex Clinic in the Psychiatry department of a medical university in Bangladesh. The participants were divided into two groups: 50 women who were seeking psychiatric consultation for sexual problems and 50 women who sought consultation for other reasons. Clinical diagnoses were established by consultant psychiatrists based on DSM-5 criteria, after which the participants were recruited by the researchers. The overall response rate was 84%, with 70% of participants coming from the outpatient department and 30% from the Psychiatric Sex Clinic. Interviews were conducted on a one-to-one basis after obtaining informed written consent from the participants. The sampling method was consecutive, and each participant completed a sociodemographic questionnaire along with the Bangla-adapted FSFI-6. The study received approval from the Institutional Review Board (IRB) of the respective university, on September 22, 2021, and the research was conducted in 2022. No compensation was provided to participants. The study funding was partially supported by the university and partially self-funded by the author.

Determining the appropriate sample size for validation studies can be challenging, with various authors offering different recommendations. Some suggest a minimum participant-to-variable ratio of 2:1 or a factor extraction ratio of 20:1.^16^ In this study, we used a more conservative approach, setting a ratio of 15 participants per item. Given that the FSFI-6 has six items, the required sample size was calculated to be 90. To account for a potential dropout rate of 10%, the target sample size was increased to 100 participants. Thus, the final sample size for this study was 100 respondents, ensuring that the ratio between the sample size and the number of items met the recommended threshold for robust validation. This sample size aligns with other validation studies, such as the work by Guendler et al. (2023)^17^ reinforcing the appropriateness of our approach.

### Study design and assessments

The study employed a cross-sectional design, in which all participants completed a semi-structured sociodemographic questionnaire along with the Bangla-adapted version of the FSFI-6. The sociodemographic questionnaire contained questions about age, educational background, occupation, residential status, family structure, duration of marital commitment, frequency of sexual activity per month, substance abuse, and the presence of pre-existing physical or mental health conditions.

### FSFI-6 Bangla version

The FSFI-6 is the abridged version of the original FSFI, a comprehensive, multidimensional self-report instrument for assessing female sexual function. The original FSFI, developed by Rosen et al. in 2000,^9^ contains 19 items that cover six core domains: desire, subjective arousal, lubrication, orgasm, satisfaction, and pain. The FSFI evaluates sexual functioning over the preceding 4 weeks, with each item scored from 0 (lowest) to 5 (highest). Lower total scores suggest a higher likelihood of sexual dysfunction. Given the extensive nature of the original FSFI, Isidori et al.10 developed the FSFI-6 in 2010 to offer a shorter, more efficient assessment tool, particularly suitable for outpatient settings. This abbreviated version retains the core elements of the FSFI, comprising six questions extracted from the original form (questions 2, 4, 7, 11, 16, and 17). It is designed to be simpler and quicker to administer, providing a practical alternative to the full FSFI-19. In the FSFI-6, the total score for each scale is calculated by summing the responses to the relevant items. The "desire" and "satisfaction" items are rated on a 5-point Likert scale (1 to 5), while the other items use a 6-point scale (0 to 5). The overall total score for the FSFI-6 ranges from 2 to 30, with lower scores indicating poorer sexual function. In the original validation study with Italian women, a score of 19 or below was used as a cutoff to classify women as having FSD. The brevity and ease of use of the FSFI-6 make it a practical tool for screening and research in outpatient and clinical settings.

### Translation and adaptation

The adaptation of the Female Sexual Function Index-6 (FSFI-6) into Bangla followed a rigorous standard procedure to ensure linguistic and cultural accuracy. After obtaining approval from the IRB and securing ethical clearance, the English version of the FSFI-6 was subjected to a multi-step translation process. First, two separate translators independently translated the English questionnaire into Bangla, resulting in two versions: T1 and T2. These versions were synthesized into a single translation, labeled T12, through a consensus process. This synthesized Bangla version was then back-translated into English by two different translators, producing two additional versions: BT1 and BT2. An expert committee comprising seven specialists with expertise in psychiatry, sexual medicine, and both English and Bangla reviewed all forward and backward translations, along with the original English version, to ensure consistency and accuracy. This committee synthesized the various translations and made linguistic adjustments, resulting in a prefinal version of the Bangla FSFI-6. The prefinal version was then pretested on 10 sexually active females to evaluate its comprehensibility and cultural appropriateness. Feedback from this pretesting phase was reviewed by the expert committee, leading to further refinements. Following this iterative process, the final Bangla version of the FSFI-6 was completed, ensuring that it was both linguistically sound and culturally relevant.

### Data processing and analysis

After data collection, the dataset was meticulously reviewed for omissions, inconsistencies, and improbabilities. The data underwent a thorough editing process to correct any errors, followed by coding to prepare for analysis. The cleaned data were then entered into a computer system for statistical analysis. Statistical analysis was carried out using the Statistical Package for Social Sciences (SPSS), version 25, in conjunction with the Classical and Bayesian Instrument Development (CBID) Beta version (accessible online at https://biostats-shinyr.kumc.edu/CBID/). The analysis focused on several key aspects to validate the Bangla adaptation of the FSFI-6. These included item-total correlation and internal consistency to assess reliability, as well as factor analysis to examine the underlying structure of the adapted questionnaire. Additionally, the receiver operating characteristic curve was utilized to determine the optimal cutoff value for the Bangla FSFI-6 in screening for positive cases of FSD. This analysis used clinically diagnosed cases of FSD as the reference outcome to evaluate the sensitivity and specificity of the cutoff point.

## Results

### Participant characteristics

The mean age of the study participants was 30 ± 5.4 years, with their husbands averaging 35.8 ± 6.5 years. Among the participants, 43% were in the 20-29–year age range, while 51% were aged between 30 and 39 years. The average duration of married life was 7.3 ± 5.9 years, and the median frequency of sexual intercourse was five times per month. Regarding privacy, 93% of the participants reported having sufficient privacy for sexual intimacy. A significant majority of the participants (77%) resided in urban areas and came from nuclear families (67%). In terms of education, 59% of the women had at least a graduation-level degree, and 48% were housewives, while 38% worked in the service sector. Additionally, 28% of the participants reported having a physical or mental illness, though none were diagnosed with psychotic disorders, and all were in stable condition at the time of the study. Notably, none of the participants reported any substance use.

### Reliability

Among the respondents, 50% reported experiencing sexual problems. The mean ± SD score for the Bangla version of the FSFI-6 across the entire sample was 18.4 ± 5.4, with scores ranging from 7 to 27. Item-total correlation analysis was employed to evaluate the relationship between individual items and the total scale score. In this analysis, the desire, lubrication, and pain items showed strong positive correlations with the total score, ranging between 0.60 and 0.79. The arousal, orgasmic function, and satisfaction items exhibited very strong positive correlations, with values exceeding 0.80 (Table 1).

**Table 1:** Distribution of item characteristics of FSFI-6 Bangla.

Item	Mean	SD	Item total correlation coefficient	Cronbach’s alpha if an item deleted
Desire	2.59	0.86	0.734	0.869
Arousal	2.71	1.02	0.893	0.839
Lubrication	3.51	1.22	0.775	0.867
Orgasmic function	2.95	1.30	0.880	0.841
Satisfaction	3.29	1.18	0.856	0.844
Pain	3.41	1.18	0.641	0.897
Total	18.46	5.46	—	—

Abbreviation: FSFI-6, Female Sexual Function Index–6.

The internal consistency of the FSFI-6, measured using Cronbach's alpha, was found to be 0.887 for the study population. A Cronbach's alpha value above 0.70 is typically considered a reliable threshold for research instruments, indicating that the internal consistency of the FSFI-6 was sufficient. Notably, removing any individual item did not significantly affect the internal consistency, supporting the decision to retain all six items in the scale.

Table 2 presents the inter-item correlations for the FSFI-6 Bangla scale. Most items demonstrated moderate to strong positive correlations with one another, with the exception of the "pain" item, which showed weak to moderate correlations with other items. Despite this, all correlations were statistically significant (p = 0.000), further reinforcing the validity of the FSFI-6 in assessing female sexual function.

**Table 2:** Correlations between the Female Sexual Function Index–6 items.

	Desire	Arousal	Lubrication	Orgasmic function	Satisfaction
Desire	1.00				
Arousal	0.614	1.00			
Lubrication	0.598	0.567	1.00		
Orgasmic function	0.536	0.787	0.603	1.00	
Satisfaction	0.630	0.759	0.565	0.807	1.00
Pain	0.301	0.558	0.368	0.421	0.368

### Validity

Face validity of FSFI-6 Bangla was established as an expert committee and naïve respondents both opined that the purpose of the test was clear and it should measure aspects of sexual function. Content validity was assessed systematically during the original instrument development. Every item of translation was assessed by Expert Committee. Confirmatory factor analysis was done by using CBID software and it revealed χ^2^ = 39.5, *P* = 0.000 and χ^2^/df as 4.39; root mean square error of approximation (RMSEA) = 0.185, comparative fit index (CFI) = 0.984, and Tucker-Lewis index (TLI) = 0.974 for the proposed one factor model. In confirmatory factor analysis (CFA), the 6th item (dyspareunia) did not fit appropriately in the proposed one factor model. If we remove that item from analysis, our model fitness improves. Confirmatory factor analysis for 5 items considered as one factor yielded χ^2^ = 11.5, df = 5, *P* = 0.000 and χ^2^/df as 2.3; RMSEA = 0.115, CFI = 0.998, and TLI = 0.995. The receiver operating characteristic (ROC) curve was utilized to assess the performance of the predictive model. The area under the curve (AUC) was found to be 0.998, at the significance level of 0.000 showing excellent discriminative ability; the cutoff value of 19 could discriminate participants with sexual disorders and no/other psychiatric diagnoses at 96% sensitivity and 100% specificity levels.

## Discussion

Given the lack of a validated tool for screening women for FSD in Bangladesh, the goal of this research was to culturally adapt and validate the FSFI-6 for Bangla-speaking women. The process began with approval from the IRB and obtaining ethical clearance. The FSFI-6 was then methodically translated and adapted into Bangla following a rigorous process. Face validity and content validity were carefully maintained during the translation and adaptation process.^18^ An expert committee comprising professionals with expertise in psychiatry, sexual medicine, and both English and Bangla assessed the translation by comparing it to the original and back-translated versions. The committee concluded that the final Bangla version of the FSFI-6 was a suitable instrument for evaluating FSD in Bangladesh. Following pretesting, the Bangla version of the FSFI-6 was finalized and prepared for data collection.

Reliability was assessed by internal consistency; all of the interitem correlations and corrected item-total correlations were within an acceptable range, confirming good internal consistency supported by Cronbach’s alpha value 0.887. The acceptable alpha value ranges from 0.70 to 0.95.^19^ Deleting any items did not bring any significant change in internal consistency, that is why all items were kept. The Cronbach alpha value was matched with other FSFI and FSFI-6 studies, i.e., alpha value: 0.82,^9^ 0.789,^10^ 0.91,^11^ 0.888,^12^ 0.84,^13^ 0.856,^14^ 0.862.^15^ The inter-item correlation was moderate to strong except the “pain” item, which showed weak to moderate correlations with other items. All the correlations were statistically significant (*P* = 0.000). In a previous study of Bangladesh,^20^ the lowest mean score of subdomains was found in the “desire” domain (3.21, indicating that the maximum number of respondents (54.34%) were suffering from desire problems. In our study, we also found the desire item has the lowest mean score (2.59).

Construct validity was assessed by confirmatory factor analysis. The CFA model fitness values indicated poor fitting for a one-factor model of six items. However, removing the sixth item (dyspareunia) from analysis improved model fitness except for RMSEA value. Out findings matched with the findings of a Brazilian study of FSFI-6 validation where the values were CFI: 0.990 andTLI: 0.980.^16^ Similar factor structure was observed with Iranian,^14^ Brazilian^13^ and Portuguese^21^ validation studies of the tool.

We have determined that the cutoff value of 19 could be used to screen FSD. Using the cutoff point 19, we have found that around 50% of female patients screened positive for FSD whereas in other studies screen-positive FSDs were 65.6% in the original study by Isidori et al.,^10^ 2010; 40.5% in Korea,^12^ 53.5% in Brazil,^13^ 51% in Turkey,^15^ and 65% in Equador.^11^ Isidori et al.^10^ also set the threshold at 19, which demonstrated a sensitivity of 0.93 and a specificity of 0.94. The South Korean validation study^12^ utilized a diagnostic cutoff score of 21 for FSD, which exhibited a sensitivity of 0.89 and a specificity of 0.86. The cutoff utilized for the Brazilian version corresponds to the computed median of total scores within the sample, which was set at 21 points.^13^

To the best of the researcher’s knowledge, this study is the first to culturally adapt and validate the FSFI-6 Bangla version. The findings align well with existing literature on the FSFI-6, indicating that this abbreviated tool can be effectively applied in clinical and research settings for screening and evaluating treatment responses for FSD. However, there are some limitations to this study. One major limitation is that the study was conducted in an outpatient setting of a psychiatric unit at a single center, focusing exclusively on premenopausal participants. This could have had an impact on the generalizability of the findings. Additionally, because the study was cross-sectional, test–retest reliability was not assessed. Another limitation is the absence of other culturally adapted tools in Bangla, which prevented the researchers from assessing concurrent and criterion validity. The lack of comparable instruments makes it challenging to fully establish the validity of the FSFI-6 in this cultural context.

## Conclusions

Often underdiagnosed, FSD is an area that requires prompt and rapid attention. The main challenge in identifying FSD is unwillingness of both patient and physician to talk about the topic. So, we need a well-validated and culturally adapted screening tool to overcome the problem. With this aim, the study was conducted and the FSFI-6 Bangla was found to show significant internal consistency (Cronbach’s alpha 0.887). Face validity, content validity, and construct validity were also satisfactory. So, the FSFI-6 Bangla version is culturally adapted and psychometrically reliable and valid tool to screen FSD in the Bangladeshi female population, including quick screening of FSD in outpatient settings.
